# Lithium response in bipolar disorder is associated with focal adhesion and PI3K-Akt networks: a multi-omics replication study

**DOI:** 10.1038/s41398-024-02811-4

**Published:** 2024-02-23

**Authors:** Anna H. Ou, Sara B. Rosenthal, Mazda Adli, Kazufumi Akiyama, Nirmala Akula, Martin Alda, Azmeraw T. Amare, Raffaella Ardau, Bárbara Arias, Jean-Michel Aubry, Lena Backlund, Michael Bauer, Bernhard T. Baune, Frank Bellivier, Antonio Benabarre, Susanne Bengesser, Abesh Kumar Bhattacharjee, Joanna M. Biernacka, Pablo Cervantes, Guo-Bo Chen, Hsi-Chung Chen, Caterina Chillotti, Sven Cichon, Scott R. Clark, Francesc Colom, David A. Cousins, Cristiana Cruceanu, Piotr M. Czerski, Clarissa R. Dantas, Alexandre Dayer, Maria Del Zompo, Franziska Degenhardt, J. Raymond DePaulo, Bruno Étain, Peter Falkai, Frederike Tabea Fellendorf, Ewa Ferensztajn-Rochowiak, Andreas J. Forstner, Louise Frisén, Mark A. Frye, Janice M. Fullerton, Sébastien Gard, Julie S. Garnham, Fernando S. Goes, Maria Grigoroiu-Serbanescu, Paul Grof, Oliver Gruber, Ryota Hashimoto, Joanna Hauser, Urs Heilbronner, Stefan Herms, Per Hoffmann, Andrea Hofmann, Liping Hou, Stephane Jamain, Esther Jiménez, Jean-Pierre Kahn, Layla Kassem, Tadafumi Kato, Sarah Kittel-Schneider, Barbara König, Po-Hsiu Kuo, Ichiro Kusumi, Nina Lackner, Gonzalo Laje, Mikael Landén, Catharina Lavebratt, Marion Leboyer, Susan G. Leckband, Carlos A. López Jaramillo, Glenda MacQueen, Mario Maj, Mirko Manchia, Cynthia Marie-Claire, Lina Martinsson, Manuel Mattheisen, Michael J. McCarthy, Susan L. McElroy, Francis J. McMahon, Philip B. Mitchell, Marina Mitjans, Francis M. Mondimore, Palmiero Monteleone, Caroline M. Nievergelt, Markus M. Nöthen, Tomas Novák, Urban Ösby, Norio Ozaki, Sergi Papiol, Roy H. Perlis, Claudia Pisanu, James B. Potash, Andrea Pfennig, Daniela Reich-Erkelenz, Andreas Reif, Eva Z. Reininghaus, Marcella Rietschel, Guy A. Rouleau, Janusz K. Rybakowski, Martin Schalling, Peter R. Schofield, K. Oliver Schubert, Thomas G. Schulze, Barbara W. Schweizer, Florian Seemüller, Giovanni Severino, Tatyana Shekhtman, Paul D. Shilling, Kazutaka Shimoda, Christian Simhandl, Claire M. Slaney, Alessio Squassina, Thomas Stamm, Pavla Stopkova, Sarah K. Tighe, Alfonso Tortorella, Gustavo Turecki, Eduard Vieta, Julia Volkert, Stephanie Witt, Naomi R. Wray, Adam Wright, L. Trevor Young, Peter P. Zandi, John R. Kelsoe

**Affiliations:** 1 Department of Psychiatry, University of California San Diego, La Jolla, CA USA; 2 Center for Computational Biology and Bioinformatics, Department of Medicine, University of California San Diego, La Jolla, CA USA; 3 Department of Psychiatry and Psychotherapy, Charité— Universitätsmedizin Berlin, Campus Charité Mitte, Berlin, Germany; 4 Fliedner Klinik Berlin, Center for Psychiatry, Psychotherapy and Psychosomatic Medicine, Berlin, Germany; 5 Department of Biological Psychiatry and Neuroscience, Dokkyo Medical University School of Medicine, Mibu, Japan; 6 Intramural Research Program, National Institute of Mental Health, National Institutes of Health, US Department of Health & Human Services, Bethesda, MD USA; 7 Department of Psychiatry, Dalhousie University, Halifax, NS Canada; 8 National Institute of Mental Health, Klecany, Czech Republic; 9 Discipline of Psychiatry, University of Adelaide, Adelaide, SA Australia; 10 Unit of Clinical Pharmacology, Hospital University Agency of Cagliari, Cagliari, Italy; 11 Department of Evolutive Biology, Ecology and Environmental Sciences, Facultat de Biologia and Institut de Biomedicina (IBUB), Universitat de Barcelona, Barcelona, Spain; 12 CIBER de Salud Mental, ISCIII, Madrid, Barcelona, Catalonia, Spain; 13 Department of Mental Health and Psychiatry, Mood Disorders Unit—Geneva University Hospitals, Geneva, Switzerland; 14 Department of Molecular Medicine and Surgery, Karolinska Institutet and Center for Molecular Medicine, Karolinska University Hospital, Stockholm, Sweden; 15 Department of Psychiatry and Psychotherapy, University Hospital Carl Gustav Carus, Medical Faculty, Technische Universität Dresden, Dresden, Germany; 16 Department of Psychiatry, University of Münster, Münster, Germany; 17 INSERM UMR-S 1144—Université Paris Cité Département de Psychiatrie et de Médecine Addictologique, AP-HP, Groupe Hospitalier Lariboisière-F Widal, Paris, France; 18 Bipolar and Depressive Disorders Unit, Institute of Neuroscience, Hospital Clinic, University of Barcelona, IDIBAPS, CIBERSAM, ISCIII, Barcelona, Catalonia Spain; 19 Neurobiological Background and Anthropometrics in Bipolar Affective Disorder, Division of Psychiatry and Psychotherapeutic Medicine, Medical University of Graz, Graz, Austria; 20 Department of Health Sciences Research, Mayo Clinic, Rochester, MN USA; 21 Department of Psychiatry and Psychology, Mayo Clinic, Rochester, MN USA; 22 The Neuromodulation Unit, McGill University Health Centre, Montreal, QC Canada; 23 Department of Psychiatry & Center of Sleep Disorders, National Taiwan University Hospital, Taipei, Taiwan; 24 Institute of Human Genetics, University of Bonn and Department of Genomics, Life & Brain Center, Bonn, Germany; 25 Human Genomics Research Group, Department of Biomedicine, University Hospital Basel, Basel, Switzerland; 26 Campus for Ageing and Vitality, Newcastle University, Newcastle upon Tyne, UK; 27 Douglas Mental Health University Institute, McGill University, Montreal, QC Canada; 28 Psychiatric Genetic Unit, Poznan University of Medical Sciences, Poznan, Poland; 29 Department of Psychiatry, University of Campinas (Unicamp), Campinas, Brazil; 30 Department of Biomedical Sciences, University of Cagliari, Cagliari, Italy; 31 Department of Psychiatry and Behavioral Sciences, Johns Hopkins University, Baltimore, MD USA; 32 Institute of Psychiatric Phenomics and Genomics (IPPG) and Department of Psychiatry and Psychotherapy, Ludwig-Maximilians-University of Munich, Munich, Germany; 33 Department of Adult Psychiatry, Poznan University of Medical Sciences, Poznan, Poland; 34 Department of Clinical Neuroscience, Karolinska Institutet and Center for Molecular Medicine, Karolinska University Hospital, Stockholm, Sweden; 35 Child and Adolescent Psychiatry Research Center, Stockholm, Sweden; 36 Mental Illness Research Theme, Neuroscience Research Australia, Sydney, NSW Australia; 37 School of Medical Sciences, University of New South Wales, Sydney, NSW Australia; 38 Pôle de Psychiatrie Générale Universitaire, Centre Hospitalier Charles Perrens, Bordeaux, France; 39 Biometric Psychiatric Genetics Research Unit, Alexandru Obregia Psychiatric Hospital, Bucharest, Romania; 40 Mood Disorders Center of Ottawa, Ottawa, ON Canada; 41 Department of Psychiatry and Psychotherapy, University Medical Center (UMG), Georg-August University Göttingen, Göttingen, Germany; 42 Department of Pathology of Mental Diseases, National Institute of Mental Health, National Center of Neurology and Psychiatry, Tokyo, Japan; 43 Inserm U955, Psychiatrie Translationnelle, Créteil, France; 44 Service de Psychiatrie et Psychologie Clinique, Centre Psychothérapique de Nancy-Laxou—Université de Lorraine, Nancy, France; 45 Laboratory for Molecular Dynamics of Mental Disorders, RIKEN Brain Science Institute, Saitama, Japan; 46 Department of Psychiatry, Psychosomatic Medicine and Psychotherapy, University Hospital Frankfurt—Goethe University, Frankfurt am Main, Germany; 47 Department of Psychiatry and Psychotherapeutic Medicine, Landesklinikum Neunkirchen, Neunkirchen, Austria; 48 Institute of Epidemiology and Preventive Medicine, National Taiwan University, Taipei, Taiwan; 49 Department of Psychiatry, Hokkaido University Graduate School of Medicine, Sapporo, Japan; 50 Institute of Neuroscience and Physiology, The Sahlgrenska Academy at the Gothenburg University, Gothenburg, Sweden; 51 Department of Medical Epidemiology and Biostatistics, Karolinska Institutet, Stockholm, Sweden; 52 Assistance Publique-Hôpitaux de Paris, Hôpital Albert Chenevier—Henri Mondor, Pôle de Psychiatrie, Créteil, France; 53 Department of Pharmacy, VA San Diego Healthcare System, La Jolla, CA USA; 54 Department of Psychiatry, University of Antioquia, Medellín, Medellín, Colombia; 55 Department of Psychiatry, University of Calgary, Calgary, AB Canada; 56 Department of Psychiatry, University of Naples SUN, Naples, Italy; 57 Department of Medical Sciences and Public Health, University of Cagliari, Cagliari, Italy; 58 Department of Pharmacology, Dalhousie University, Halifax, NS Canada; 59 Department of Clinical Neurosciences, Karolinska Institutet, Stockholm, Sweden; 60 Department of Biomedicine, Aarhus University, Aarhus, Denmark; 61 Department of Psychiatry, VA San Diego Healthcare System, La Jolla, CA USA; 62 Department of Psychiatry, Lindner Center of Hope, University of Cincinnati, Mason, OH USA; 63 School of Psychiatry, University of New South Wales, and Black Dog Institute, Sydney, NSW Australia; 64 Department of Genetics, Microbiology, and Statistics, Faculty of Biology and Institut de Biomedicina (IBUB), Universitat de Barcelona, Barcelona, CIBER de Salud Mental, ISCIII, Madrid, Spain; 65 Neurosciences Section, Department of Medicine and Surgery, University of Salerno, Salerno, Italy; 66 Department of Neurobiology, Care Sciences, and Society, Karolinska Institutet and Center for Molecular Medicine, Karolinska University Hospital, Stockholm, Sweden; 67 Department of Psychiatry, Nagoya University Graduate School of Medicine, Nagoya, Japan; 68 Ludwig-Maximilians-University of Munich, Munich, Germany; 69 Department of Psychiatry, Massachusetts General Hospital and Harvard Medical School, Boston, MA USA; 70 Department of Psychiatry, University of Iowa, Iowa City, IA USA; 71 Department of Genetic Epidemiology in Psychiatry, Central Institute of Mental Health, Medical Faculty Mannheim, University of Heidelberg, Mannheim, Germany; 72 Montreal Neurological Institute and Hospital, McGill University, Montreal, QC Canada; 73 Veterans Administration, San Diego Healthcare System, San Diego, CA USA; 74 Department of Psychiatry, Dokkyo Medical University School of Medicine, Mibu, Japan; 75 Bipolar Center, Wiener Neustadt, Austria; 76 University of Iowa Carver College of Medicine and University of Iowa College of Public Health, VA Quality Scholars Program, Iowa City VA Hospital, Iowa City, IA USA; 77 The University of Queensland, Queensland Brain Institute, Brisbane, QLD Australia; 78 Department of Psychiatry, University of Toronto, Toronto, ON Canada; 79 Department of Mental Health, Johns Hopkins Bloomberg School of Public Health, Baltimore, MD USA

**Keywords:** Clinical genetics, Bipolar disorder

## Abstract

Lithium is the gold standard treatment for bipolar disorder (BD). However, its mechanism of action is incompletely understood, and prediction of treatment outcomes is limited. In our previous multi-omics study of the Pharmacogenomics of Bipolar Disorder (PGBD) sample combining transcriptomic and genomic data, we found that focal adhesion, the extracellular matrix (ECM), and PI3K-Akt signaling networks were associated with response to lithium. In this study, we replicated the results of our previous study using network propagation methods in a genome-wide association study of an independent sample of 2039 patients from the International Consortium on Lithium Genetics (ConLiGen) study. We identified functional enrichment in focal adhesion and PI3K-Akt pathways, but we did not find an association with the ECM pathway. Our results suggest that deficits in the neuronal growth cone and PI3K-Akt signaling, but not in ECM proteins, may influence response to lithium in BD.

## Introduction

Bipolar disorder (BD) is a chronic psychiatric illness that presents with episodes of mania, depression, and sometimes psychosis. Globally, it is the sixth leading cause of medical disability among people from 15 to 44 years old. Patients with BD are at a higher risk of suicide than those with any other psychiatric or medical illness. Some studies report that roughly 50% of patients will attempt suicide, and up to 20% of untreated patients will complete suicide [1], while treatment by lithium reduces that risk significantly [2, 3]. Unfortunately, misdiagnosis is common and often delays an accurate treatment. Up to 70% of patients are initially misdiagnosed, usually with major depressive disorder. On average, there is a delay of 8 years before the correct diagnosis of BD is made [4]. During this time, patients continue to suffer, may be treated with medications that make their illness course worse, and are at risk of suicide.

Lithium is the gold standard treatment for BD [5]. Its mechanism of action is still not completely understood [6]. Many studies have investigated the neurotrophic effect of lithium. One theory posits that chronic administration of lithium inhibits glycogen synthase kinase 3 (GSK3β), a serine/threonine kinase. This leads to anti-apoptotic effects and improved cell structural stability [7–10]. GSK3β has also been shown to exhibit interactions with many pathways, including phosphorylation of several components of the PI3K/AKT/mTOR signaling network, as well as regulation of transcription for proteins bound to microtubules [11]. Another theory involves the phosphoinositol (PI) cycle. In the PI cycle, lithium inhibits inositol monophosphatase, which ultimately downregulates protein kinase C isozymes such as myristoylated alanine-rich C-kinase substrate (MARCKS). MARCKS is an actin-binding protein found in neuronal processes that is implicated in cytoskeletal restructuring. Its downregulation stabilizes the neuronal membrane and results in neurotrophic effects [7, 12]. A more recent theory proposes that lithium alters the phosphorylation state of collapsin response mediator protein-2 (CRMP2). CRMP2 regulates cytoskeletal organization, particularly in dendritic spines [13, 14]. Finally, a study using polygenic score modeling has indicated that the cholinergic and glutamatergic pathways may potentially serve as targets for lithium [15]. It is possible that lithium exerts its effects through multiple or all of these pathways. A single definitive model remains elusive, but interactions with neuronal cytoskeleton are possibly involved.

Interestingly, there is a range of responses to treatment with lithium. Previous studies have reported that 20–30% of patients with BD are excellent responders, whereas over 40% fail to demonstrate any significant clinical improvement. These patient populations have been shown to differ from each other both phenotypically and genetically [16]. A differential response to lithium has been previously demonstrated between induced pluripotent stem cell (iPSC) neurons derived from lithium responders and non-responders. The hyperexcitability of in vitro neurons derived from BD patients was reversed by lithium treatment, but only in those from patients who were lithium responders [17]. This finding is also supported by family studies, which found that the relatives of lithium responders were significantly more likely to be lithium responders as well [18, 19]. These studies imply that patients with BD could be subcategorized based on biological differences which induce a divergent lithium response. There is a great need to better understand these differences in order to identify possible predictors of treatment response. However, dozens of previous candidate-gene association studies, genome-wide association studies (GWAS), and polygenic risk score analyses of lithium response in BD have failed to identify genetic variants with major effects. Given this pressing need to find pharmacogenetic predictors of response, more advanced methods in integrative genomic analysis are necessary [16].

GWAS inherently face several limitations when used in isolation, including the challenge of genetic heterogeneity. In many disease processes with genetic associations, patients may carry diverse combinations of causal variants that impact multiple genes, creating a net effect across a particular pathway. GWAS of BD primarily detect variants of very small effect size consistent with a polygenic mode of transmission. Since each single nucleotide polymorphism (SNP) contributes only a tiny amount to the overall predisposition to BD, enormous sample sizes are required, and it can be difficult to surmise mechanisms of disease. Network approaches seek to address this biological reality by integrating GWAS results with known protein-protein interactions and other molecular networks. New causal genes may be identified by boosting their interactions with products of known causal genes [20, 21].

We have recently reported a combined analysis of transcriptomic and GWAS data from the Pharmacogenomics of Bipolar Disorder (PGBD) study [22] of treatment response to lithium. After using network propagation to reprioritize candidate genes from GWAS data, we found significant overlap between both transcriptomic and GWAS results. The joint analysis yielded a 500 gene network significantly enriched in the following Kyoto Encyclopedia of Genes and Genomes (KEGG) pathways: focal adhesion, ECM-receptor interaction, and PI3K-Akt signaling [23]. All three pathways play a role in axon growth and neuronal development [24]. Consistent with these results, post-mortem studies have found that in BD, neuronal populations may exhibit a decrease in number, size, and/or amount of dendritic spines [13, 25]. Given that lithium may have downstream effects on these pathways, it is possible that genetic defects in focal adhesion pathways may provide both a mechanism for susceptibility to BD as well as a target for lithium treatment.

In this study, we aimed to replicate the results of our previous multi-omics study on a larger dataset of over 2000 patients from the International Consortium on Lithium Genetics (ConLiGen) [26]. We reprioritized GWAS results using network methods to determine overlap with focal adhesion, ECM-receptor interaction, and PI3K-Akt signaling pathways.

## Methods

Summary statistics were downloaded from the NHGRI-EBI GWAS Catalog [27] on 12/12/2022 for study GCST012487 [26]. The data resulted from a GWAS of lithium response in 2563 patients at 22 sites participating in the International Consortium on Lithium Genetics (ConLiGen). We utilized the summary statistics from a combined sample of 2039 European ancestry individuals. In the ConLiGen study, data from over 6 million single nucleotide polymorphisms (SNPs) were tested for association with categorical and continuous retrospective ratings of lithium response using the Alda scale [28, 29]. The Alda scale includes two scores: score A is a 0–10 retrospective rating of lifetime response, while score B captures factors reducing the confidence in score A such as lack of a documented lithium level, etc. In the ConLiGen study, under the continuous phenotype, participants were rated with the Alda A score, and individuals with a B score greater than 4 were excluded. We used the continuous rather than the dichotomous phenotype as a measure of treatment response because genome-wide significant association was detected with the continuous phenotype in the original GWAS. Quality control and statistical analysis methods are described in the original paper.

### SNP, gene, and gene-set analysis

We imported the ConLiGen summary statistics into FUMA (Functional Mapping and Annotation of Genome-Wide Association Studies—https://fuma.ctglab.nl) [30], a web-based platform for annotating, prioritizing, visualizing and interpreting GWAS results. We utilized the SNP2GENE function to map SNPs to genes and conduct SNP, gene-based, and gene-set analysis. We used all default settings, except for setting the maximum lead SNP *p* value to 1 × 10e−5.

### Network analysis

We input the ConLiGen summary statistics into NAGA (Network Assisted Genomic Analysis), an online network propagation tool for pathway boosting and interpretation of genome-wide association studies [21]. NAGA provided a reprioritized ranked list of 19,781 genes as output. We then entered the top 500 genes with the highest final heat scores into STRING, an online database that generates mapped networks based on protein-protein interactions [31]. STRING additionally analyzes for overrepresentation of user-inputted gene lists in established pathways, using the hypergeometric test [32]. Using this function, we tested our a priori hypotheses to identify functional enrichment of the NAGA-generated top 500 gene list in the KEGG hsa04510 focal adhesion pathway, KEGG hsa04512 ECM-receptor interaction, and KEGG hsa04151 PI3K-Akt signaling pathway [33]. *p* values were corrected for multiple testing by STRING using the Benjamini–Hochberg procedure [34].

Overlap between the NAGA-generated top 500 gene list and the KEGG pathways was visualized using Cytoscape [35]. A hypergeometric test was conducted to test for overrepresentation of the NAGA-generated 500 gene network in the 500 gene network generated in our previous study [23].

## Results

### Demographics

The demographics of the sample can be found in the original ConLiGen study [26]. The study was conducted in two phases: GWAS 1 (*n* = 1065) and GWAS 2 (*n* = 1168). Sex and age were similar across both cohorts. Mean Alda scale A scores were 6.13 (SD = 3.13) and 6.52 (SD = 2.87), respectively. Mean Alda scale B scores were 1.78 (SD = 1.26) and 2.35 (SD = 1.16), respectively.

### SNP, gene, and gene-set analysis

As reported in the original ConLiGen study, the only SNPs that were significant at a genome-wide significance level of 5e-08 were in linkage disequilibrium with the SNP rs74795342 on chromosome 21 (Supplementary Fig. 1). Using FUMA in our gene-wise analysis, no significant genes were found at a significance level of *p* < 0.05/18314 = 2.730e−6 (Supplementary Fig. 2). No gene-sets were found to be significant either, using *p* < 0.05 after Bonferroni correction. The most highly associated genes and gene-sets are listed in Supplementary Tables 1 and 2.

### Network analysis

**Fig. 1:**
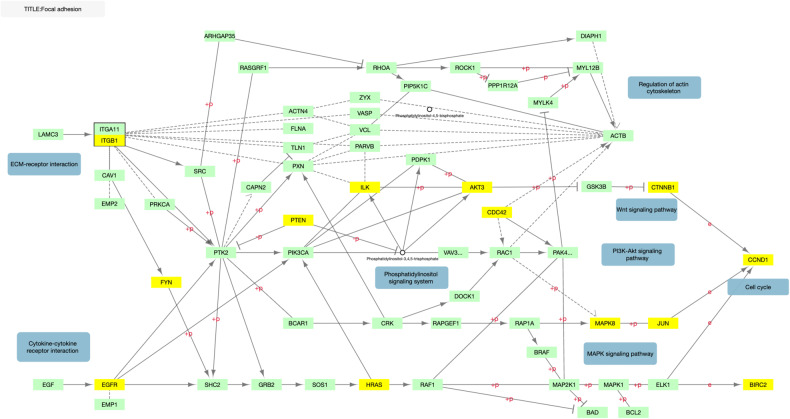
Overlap between KEGG focal adhesion and top 500 genes. KEGG hsa04510 pathway for focal adhesion adapted to illustrate gene overlap. Genes in yellow overlap with the 500 gene NAGA network.

**Fig. 2:**
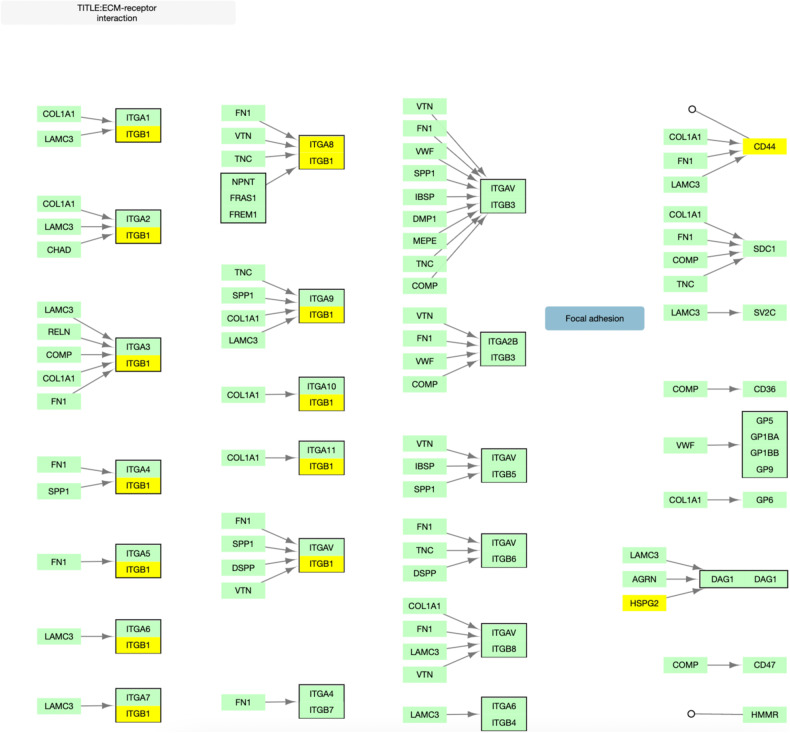
Overlap between KEGG ECM-receptor interaction and top 500 genes. KEGG hsa04512 pathway for ECM-receptor interaction adapted to illustrate gene overlap. Genes in yellow overlap with the 500 gene NAGA network.

**Fig. 3:**
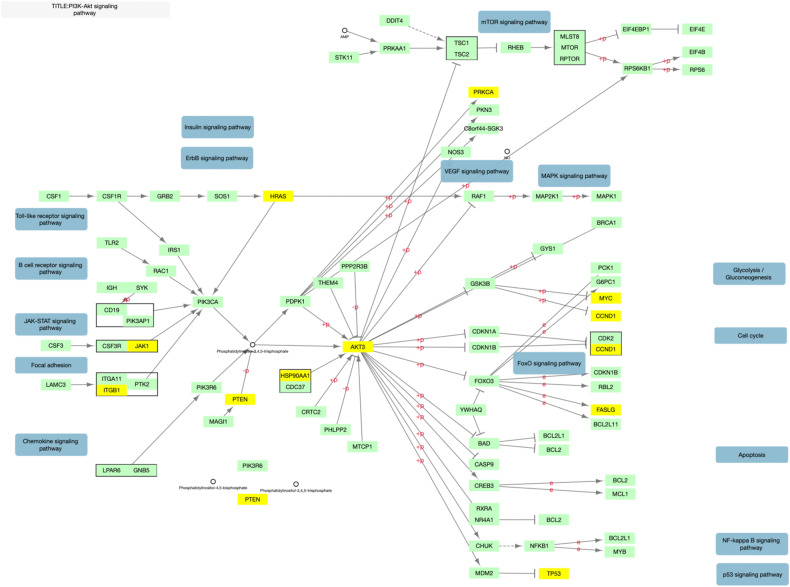
Overlap between KEGG PI3k-Akt and top 500 genes. KEGG hsa04151 pathway for PI3k-Akt signaling adapted to illustrate gene overlap. Genes in yellow overlap with the 500 gene NAGA network.

We first tested the three a priori pathways that were significant in our previous study, which had examined an independent sample [23]. Using the STRING analysis function, the top 500 reprioritized gene list generated by NAGA was found to be significantly enriched in both the KEGG hsa04510 focal adhesion pathway (*p* = 1.74e−06) and KEGG hsa04151 PI3K-Akt signaling pathway (*p* = 1.90e−07) (Table 1). Given the goal of replication and the small number of statistical tests, this was considered as a significant replication of our previous results in an independent sample for the focal adhesion and PI3K-Akt pathways. However, the KEGG hsa04512 ECM-receptor interaction pathway was not found to be significantly enriched (Table 1). The overlapping genes in all three networks can be seen in Figs. 1–3.

**Table 1:** Functional enrichment of NAGA top 500 gene list in focal adhesion, ECM, and PI3K-Akt pathways.

Pathway	*p* value	Number of genes overlapped
KEGG focal adhesion	1.74e−06*	21 of 198
KEGG ECM-receptor interaction	0.1494	5 of 88
KEGG PI3k-Akt	1.90e−07*	31 of 350

All *p* values corrected for multiple testing using the Benjamini–Hochberg procedure.

*Significant at *p* < 0.05.

A hypergeometric test found significant overlap (*p* = 5.699e−07) between the 500 gene network generated by NAGA and the 500 gene network generated by network propagation analysis in our previous study [23]. There were 33 genes that were common to both networks. The top 25 reprioritized genes produced by NAGA are listed in Table 2. All top 500 reprioritized NAGA genes are listed in Supplementary Table 4.

**Table 2:** NAGA top 25 gene list.

NAGA				FUMA gene-wise analysis	
Gene	Input heat	Final heat	Rank	Rank	*p* value
UBC	0	37.88310418	1	4335	0.22849
GNB1	2.624306658	21.36254772	2	12993	0.69873
PRKACB	6.770605441	19.12717575	3	5778	0.30963
GNAL	0	18.71021909	4	16294	0.88169
GNGT1	0	18.61047924	5	12956	0.69712
REEP1	0	17.83396241	6	13325	0.71728
ARRB1	7.336233161	17.55148794	7	12430	0.66845
RTP2	0	17.50819164	8	11202	0.60084
RTP1	0	17.50727661	9	13284	0.71484
PRKACA	0	15.7274719	10	5506	0.29380
ARRB2	0	15.43877781	11	14216	0.76615
PRKACG	0	15.38554785	12	10721	0.57518
GRK2	0	15.30495263	13	^a^	^a^
GNG13	0	14.28098978	14	9444	0.50737
GNG7	0	14.26968321	15	2636	0.13696
GRK3	0	13.36097413	16	^a^	^a^
TAF1	0	10.51679188	17	^a^	^a^
APP	0	9.571290241	18	11903	0.63889
JUN	5.596453897	9.123060759	19	6091	0.32547
HNF4A	0	7.372223527	20	13583	0.73157
ELAVL1	0	7.080313649	21	2194	0.1154
C1orf94	14.45796462	7.001838181	22	9155	0.49203
CSMD2	14.45796462	6.383962772	23	36	0.0016402
KCNJ5	11.85176049	6.256678752	24	13972	0.75419
INS	3.272540349	5.764244643	25	10662	0.57147

^a^Data does not exist as gene was not evaluated in FUMA.

After testing the three a priori hypotheses based on previous results, we tested the top 500 NAGA gene list for enrichment in all pathways in STRING. The top 10 KEGG pathways found to be most strongly enriched are found in Supplementary Table 3. These include cancer and growth pathways (such as Pathways in Cancer, Estrogen Signaling Pathway, Ras Signaling Pathway) as well as the dopaminergic synapse pathway.

We additionally used the STRING analysis function to test for functional enrichment of the top 100, 200, 300, and 400 reprioritized gene lists generated by NAGA in all three a priori KEGG pathways. The results agreed with the primary analysis, since all gene lists were significantly enriched in the KEGG hsa04510 focal adhesion pathway and KEGG hsa04151 PI3K-Akt signaling pathways at a level of *p* < 0.05. Only the top 100 reprioritized gene list was found to be significantly enriched in the KEGG hsa04512 ECM-receptor interaction pathway (*p* = 0.0050) inconsistent with a robust result. (Supplementary Table 5).

## Discussion

In this study, we attempted to replicate our previous results which were from an independent sample [23]. We used network methods via NAGA to reprioritize GWAS results from the ConLiGen study on lithium response and used STRING to test three a priori network hypotheses: KEGG focal adhesion, ECM-receptor interaction and PI3K-Akt signaling. Two of these three networks, KEGG focal adhesion and PI3K-Akt signaling, were enriched in our top 500 reprioritized genes. However, we did not find significant enrichment for the ECM-receptor interaction pathway in the 500 gene network. Besides this pathway, we were otherwise able to replicate the results of our previous paper in a larger, independent sample of patients with BD. We found highly significant overlap between the top 500 gene network generated by NAGA in this study and the 500 gene network generated in the previous study, providing further evidence for replication.

Focal adhesions are points of contact between cells and proteins in the ECM. The formation of cell-ECM adhesion structures is initiated by cell surface integrins and driven by local actin polymerization. These structures function to not only mediate cell attachment to ECM, but also mediate transmembrane signaling. Integrin-ECM ligand binding can induce a number of downstream changes affecting cell shape, growth, and proliferation [36]. In neurons, specifically, the actin cytoskeleton of growth cones interacts with the ECM to guide axon development and extension [24, 37].

We had originally hypothesized that genetic deficits in focal adhesion, ECM, and PI3K-Akt pathways may impair axonal growth in neurons and determine response to lithium. Though one integrin protein was included in our top 500 genes, in general ECM proteins did not overlap with the top 500 gene list (Supplementary Table 4) (Fig. 2), and the pathway was not significant. This result is inconsistent with our previous study. However, it may suggest the possibility that the deficits influencing lithium response may be inherent to the growth cone rather than components of the ECM. This is supported by a number of studies, which have shown that lithium prevents collapse and induces growth of growth cones [38–40].

Previously, neurons derived from induced pluripotent stem cells of patients with BD have been shown to exhibit hyperexcitability in vitro. This hyperexcitable phenotype was rescued by lithium only in neurons derived from lithium good responders [17]. Elevated neuroactivity in BD may induce vulnerability in neurons through impairment of focal adhesion pathways. Chronic elevation of neuroactivity has been shown to dramatically reduce surface expression of integrin β1 in animal models, leading to axonal and dendritic degeneration and eventually cell death [41].

Unsurprisingly, neurons in patients with BD have been shown to be present with smaller size, fewer numbers, and more limited branching. We had previously proposed that in lithium responders, this deficit is caused by deficits in focal adhesion and is rescued by lithium treatment. Furthermore, we proposed that in patients who are not lithium responders, focal adhesion is not dysregulated, and lithium is unable to address the relevant impairments [42–44]. Our results in this study are consistent with this hypothesis.

After testing our three a priori hypotheses, we conducted exploratory analyses using network methods. We listed the top 10 most significant KEGG pathways that were associated by STRING with the NAGA generated gene list in Supplementary Table 3. These pathways are mostly cancer pathways associated with cell growth and proliferation or pathways of addiction and other dopamine-related processes. Dopamine neurotransmission has previously been associated with response to lithium treatment in BD [45]. Genes in associated cancer pathways show some overlap with focal adhesion as well, which suggests the possibility of shared mechanisms (Fig. 1).

Limitations of our study include the relatively small sample size (*N* = 2039) and the generalizability of the dataset, given that all participants were of European descent. Additionally, data was collected retrospectively. As a result, outcomes may be less accurate in determining response phenotypes [46] which can blur our findings due to false negatives.

This study also demonstrates the utility of network propagation methods, which can add power to GWAS with limited sample sizes. These methods are beneficial in identifying which genes and gene-sets are of interest to a disease process, but future research is still indicated for confirmation [20, 21].

In summary, we replicated our previous results reinforcing that genetic deficits in focal adhesion and PI3K-Akt signaling are associated with lithium response in BD patients. We hypothesize, as before, that malformed axonal growth cones result in shorter and less branched axons and susceptibility to BD in a subpopulation of patients who are lithium responders. This is also consistent with the idea that response to lithium results from a disease mechanism distinct from that of lithium non-responders. Furthermore, we propose that lithium rescues disrupted neuronal growth and axon extension processes by addressing deficits in focal adhesion. A better understanding of the pathophysiology of BD and lithium treatment may lead to the future development of drugs similar to lithium, as well as possible clinical predictors for treatment response.

### Supplementary information

Supplemental

Figure 1 (Supplemental)

Figure 2 (Supplemental)

## Data Availability

Summary statistics used in this study are available through the NHGRI-EBI GWAS Catalog as study number GCST012487.
