# Supplementary material for: Lithium response in bipolar disorder is associated with focal adhesion and PI3K-Akt networks: a multi-omics replication study
Source: Transl Psychiatry. 2024 Feb 23;14:109. doi: 10.1038/s41398-024-02811-4 (PMC10891068; doi:10.1038/s41398-024-02811-4)
Supplement: Supplementary file 1 — Supplemental [file 41398_2024_2811_MOESM1_ESM.docx]

#### **SUPPLEMENT**

###### **Figure 1. Manhattan Plot (GWAS Summary Statistics)**

Genome-wide significant association was defined at *p* < 5e–8 (red dashed line in the plot). SNPs above the dashed line were in linkage disequilibrium with the SNP rs74795342 on chromosome 21.

###### **Figure 2. Manhattan Plot (Gene-Based Test)**

Input SNPs were mapped to 18314 protein coding genes. Genome wide significance (red dashed line in the plot) was defined at *p* < 0.05/18314 = 2.730e-6.

###### **Table 1. Top Nominally Significant Genes Identified by MAGMA Gene Analysis through FUMA**

| Gene | P-value |
| --- | --- |
| ENSG00000048052 | 5.44E-07 |
| ENSG00000111218 | 2.97E-06 |
| ENSG00000120471 | 7.13E-06 |
| ENSG00000121904 | 5.26E-07 |
| ENSG00000144455 | 8.74E-06 |
| ENSG00000150636 | 7.11E-06 |
| ENSG00000166479 | 8.90E-05 |
| ENSG00000175928 | 8.74E-06 |
| ENSG00000186487 | 5.98E-06 |
| ENSG00000200553 | 9.91E-06 |
| ENSG00000201148 | 1.48E-05 |
| ENSG00000224935 | 3.44E-05 |
| ENSG00000226204 | 3.00E-09 |
| ENSG00000232193 | 1.25E-08 |
| ENSG00000250183 | 9.91E-06 |
| ENSG00000250392 | 9.91E-06 |
| ENSG00000250999 | 3.89E-06 |
| ENSG00000272727 | 4.15E-06 |

######

###### **Table 2. Top 10 Gene-Sets Identified by MAGMA Gene-Set Analysis through FUMA**

| Gene-Set | N genes | P-value | P*_bon_* |
| --- | --- | --- | --- |
| Curated_gene_sets:bruins_uvc_response_via_tp53_group_c | 85 | 4.23E-05 | .654229968 |
| Curated_gene_sets:kegg_arachidonic_acid_metabolism | 58 | 7.24E-05 | 1 |
| GO_bp:go_negative_regulation_of_vascular_endothelial_cell_proliferation | 4 | .0002169 | 1 |
| GO_bp:go_vascular_associated_smooth_muscle_cell_apoptotic_process | 9 | .00027558 | 1 |
| GO_bp:go_positive_regulation_of_g_protein_coupled_receptor_signaling_pathway | 29 | .00034482 | 1 |
| Curated_gene_sets:biocarta_acetaminophen_pathway | 5 | .00035008 | 1 |
| Curated_gene_sets:reactome_regulation_of_cytoskeletal_remodeling_and_cell_spreading_by_ipp_complex_components | 7 | .00035714 | 1 |
| Curated_gene_sets:liu_vav3_prostate_carcinogenesis_dn | 15 | .00040143 | 1 |
| GO_cc:go_intercalated_disc | 49 | .00041748 | 1 |
| Curated_gene_sets:parent_mtor_signaling_up | 525 | .00041792 | 1 |

###### **Table 3. Top 10 KEGG Pathways Significantly Enriched in Top 500 NAGA Gene List**

| Pathway | P-value | Count |
| --- | --- | --- |
| Pathways in Cancer | 6.15e-13 | 51 of 517 |
| Proteoglycans in Cancer | 3.60e-10 | 28 of 196 |
| Ras Signaling Pathway | 1.05e-09 | 29 of 226 |
| Relaxin Signaling Pathway | 1.76e-09 | 22 of 128 |
| Human Cytomegalovirus Infection | 1.76e-09 | 28 of 218 |
| Cholinergic Synapse | 4.05e-09 | 20 of 110 |
| Dopaminergic Synapse | 6.32e-09 | 21 of 128 |
| Estrogen Signaling Pathway | 1.04e-08 | 21 of 133 |
| MAPK Signaling Pathway | 1.61e-08 | 30 of 288 |
| Human Papillomavirus Infection | 5.27e-08 | 31 of 325 |

All p-values corrected for multiple testing using the Benjamini–Hochberg procedure.

######

###### **Table 4. Top 500 NAGA Reprioritized Ranked Genes**

| Gene List | Initial Heat | Final Heat |
| --- | --- | --- |
| *UBC* | 0 | 37.88310418 |
| *GNB1* | 2.624306658 | 21.36254772 |
| *PRKACB* | 6.770605441 | 19.12717575 |
| *GNAL* | 0 | 18.71021909 |
| *GNGT1* | 0 | 18.61047924 |
| *REEP1* | 0 | 17.83396241 |
| *ARRB1* | 7.336233161 | 17.55148794 |
| *RTP2* | 0 | 17.50819164 |
| *RTP1* | 0 | 17.50727661 |
| *PRKACA* | 0 | 15.7274719 |
| *ARRB2* | 0 | 15.43877781 |
| *PRKACG* | 0 | 15.38554785 |
| *GRK2* | 0 | 15.30495263 |
| *GNG13* | 0 | 14.28098978 |
| *GNG7* | 0 | 14.26968321 |
| *GRK3* | 0 | 13.36097413 |
| *TAF1* | 0 | 10.51679188 |
| *APP* | 0 | 9.571290241 |
| *JUN* | 5.596453897 | 9.123060759 |
| *HNF4A* | 0 | 7.372223527 |
| *ELAVL1* | 0 | 7.080313649 |
| *C1orf94* | 14.45796462 | 7.001838181 |
| *CSMD2* | 14.45796462 | 6.383962772 |
| *KCNJ5* | 11.85176049 | 6.256678752 |
| *INS* | 3.272540349 | 5.764244643 |
| *MYC* | 0 | 5.731856652 |
| *PPA1* | 10.18770895 | 5.637840644 |
| *HDAC1* | 5.294126162 | 5.576905519 |
| *NTRK1* | 5.179113119 | 5.570175817 |
| *ARHGAP32* | 11.85176049 | 5.551487567 |
| *PARP1* | 6.328897245 | 5.464938566 |
| *HSPA8* | 3.026603937 | 5.447260593 |
| *TP53AIP1* | 11.85176049 | 5.375239112 |
| *C11orf45* | 11.85176049 | 5.228347574 |
| *SLC2A1* | 7.511793284 | 5.194604606 |
| *NPFFR1* | 10.18770895 | 5.130296045 |
| *ACTA2* | 6.773224386 | 5.107600543 |
| *KIF20B* | 10.04851332 | 5.086133885 |
| *CCT3* | 4.880660218 | 5.06988279 |
| *NR5A2* | 10.70332947 | 5.067030424 |
| *SHOC2* | 7.537739885 | 5.063148662 |
| *MORN1* | 10.19010353 | 5.049843414 |
| *SAR1A* | 10.18770895 | 5.030099147 |
| *OR52A5* | 9.695011038 | 5.007998029 |
| *OR52A1* | 9.695011038 | 5.000662823 |
| *SPATA6* | 10.49624656 | 4.941104255 |
| *TP53* | 0 | 4.924546687 |
| *ITGB1* | 7.833086756 | 4.907312779 |
| *FAM171A1* | 10.20351215 | 4.883900726 |
| *SLC5A9* | 10.49624656 | 4.84705049 |
| *OPCML* | 9.58169395 | 4.841997976 |
| *SKI* | 10.19010353 | 4.836109417 |
| *DLG2* | 8.726064083 | 4.824449977 |
| *ESR1* | 0 | 4.8218198 |
| *RCC1* | 8.314252347 | 4.821272316 |
| *MYOD1* | 3.289834914 | 4.788296321 |
| *CREB3L1* | 7.661502082 | 4.779547333 |
| *CXCL12* | 7.304359759 | 4.766327928 |
| *KCND3* | 9.093446621 | 4.703513754 |
| *PDE4B* | 8.065162614 | 4.670875666 |
| *TYSND1* | 9.988609806 | 4.655298073 |
| *RPS3* | 7.336233161 | 4.622669814 |
| *OR52E2* | 9.695011038 | 4.62125859 |
| *PRPF19* | 7.997506939 | 4.600853685 |
| *LBR* | 7.002835485 | 4.588355766 |
| *CACNA1E* | 8.006167588 | 4.548460588 |
| *RCOR3* | 8.704125361 | 4.519297741 |
| *NXF1* | 2.420243136 | 4.513110061 |
| *TNR* | 7.202797706 | 4.49894127 |
| *ABL2* | 8.806877267 | 4.493332515 |
| *AGL* | 7.470400651 | 4.477496918 |
| *OR52J3* | 9.695011038 | 4.468338968 |
| *LRRC20* | 9.897505481 | 4.452454111 |
| *CTSD* | 6.759335274 | 4.444255063 |
| *SP1* | 0 | 4.438918125 |
| *MOV10* | 4.374852431 | 4.431211613 |
| *H2AFY2* | 9.441894191 | 4.418333167 |
| *ITGA8* | 8.706539363 | 4.410873215 |
| *APOA1* | 5.866066138 | 4.408002051 |
| *SMCP* | 3.459992428 | 4.397766702 |
| *AKT3* | 6.646930661 | 4.384954434 |
| *NRG3* | 9.493233928 | 4.379721823 |
| *FMOD* | 6.07745826 | 4.374574876 |
| *NEURL1* | 7.399595603 | 4.357269175 |
| *DDI1* | 8.137045891 | 4.35609218 |
| *AIFM2* | 9.520949949 | 4.339994146 |
| *ABTB2* | 9.666519995 | 4.336809424 |
| *PARVA* | 8.507242861 | 4.336389419 |
| *TMEM109* | 7.997506939 | 4.335452268 |
| *EGFR* | 0 | 4.329829684 |
| *XPO1* | 0 | 4.32253048 |
| *CD44* | 5.366168033 | 4.319698029 |
| *HTR3A* | 6.648472681 | 4.305388873 |
| *NTSR2* | 0 | 4.300859182 |
| *PPP1CA* | 2.701571235 | 4.253551988 |
| *GLUD1* | 7.225346882 | 4.245716435 |
| *SLC15A3* | 7.997506939 | 4.234858613 |
| *NGF* | 7.330264822 | 4.227828513 |
| *TRAF5* | 8.704125361 | 4.22559742 |
| *DNAH14* | 9.424771983 | 4.221737233 |
| *ZBTB18* | 7.926909646 | 4.2112674 |
| *DGKZ* | 7.661502082 | 4.200273014 |
| *HIST3H3* | 6.387771717 | 4.192809351 |
| *LCK* | 5.294126162 | 4.189104997 |
| *PRELP* | 5.996678114 | 4.175871424 |
| *FEN1* | 5.727563003 | 4.167782832 |
| *FMO4* | 4.992304337 | 4.166477973 |
| *NRAS* | 5.204098691 | 4.165892705 |
| *F2* | 4.649749229 | 4.162550536 |
| *LAPTM5* | 6.671892955 | 4.159929893 |
| *DAB1* | 7.247151787 | 4.155159527 |
| *FCHSD2* | 8.66976179 | 4.152866845 |
| *MMP26* | 7.699397706 | 4.152580286 |
| *LMOD1* | 7.881410328 | 4.148458983 |
| *FAS* | 6.773224386 | 4.146873032 |
| *FAM20B* | 8.806877267 | 4.135386341 |
| *CDC42* | 3.499913355 | 4.130305736 |
| *RAB4A* | 7.364250558 | 4.123508279 |
| *TIMM17A* | 7.881410328 | 4.10630995 |
| *PRSS23* | 8.258682496 | 4.100914591 |
| *TOR3A* | 8.806877267 | 4.083421142 |
| *IPO9* | 7.881410328 | 4.081089543 |
| *PAX2* | 6.633158446 | 4.079718948 |
| *HSPA6* | 4.350527968 | 4.076212181 |
| *ZBTB16* | 7.708933204 | 4.072986502 |
| *CAPZB* | 6.663241702 | 4.070701235 |
| *C1orf127* | 9.17208166 | 4.068477919 |
| *CUL5* | 5.757183251 | 4.060700648 |
| *CYR61* | 7.226034025 | 4.059942585 |
| *TAF12* | 7.980407466 | 4.049713858 |
| *OR9I1* | 8.282911896 | 4.046714438 |
| *PRKG1* | 7.156857869 | 4.044329034 |
| *GNAI3* | 4.588313069 | 4.041866682 |
| *AJAP1* | 5.443343139 | 4.041790761 |
| *KANK4* | 8.808883285 | 4.039646156 |
| *VCAM1* | 5.139605675 | 4.038140363 |
| *ANK3* | 6.896815339 | 4.037863432 |
| *CD55* | 6.402143222 | 4.035336299 |
| *PANK1* | 8.759901898 | 4.031990359 |
| *L1TD1* | 8.808883285 | 4.030518219 |
| *CCDC86* | 7.997506939 | 4.028606555 |
| *CR1* | 6.804296571 | 4.022554381 |
| *ABCC8* | 5.261214279 | 4.012429678 |
| *ATP5C1* | 6.153983477 | 4.006285274 |
| *EDEM3* | 7.657261193 | 4.004681643 |
| *COPB1* | 5.546266037 | 4.001045747 |
| *PTGDR2* | 7.997506939 | 3.99580183 |
| *CASP1* | 5.390432655 | 3.991952875 |
| *ADRA2A* | 7.652406302 | 3.987192524 |
| *NEK2* | 6.735484058 | 3.98405707 |
| *AGBL4* | 8.866750668 | 3.980519819 |
| *RET* | 3.879555815 | 3.966298198 |
| *CADM1* | 8.363185379 | 3.963259603 |
| *HNRNPF* | 5.716867714 | 3.957769032 |
| *PKN2* | 6.178758446 | 3.954009478 |
| *WNT11* | 6.186020642 | 3.951619191 |
| *PTPN7* | 7.612570264 | 3.951061608 |
| *INSRR* | 5.179113119 | 3.93540409 |
| *DEPDC7* | 8.517193191 | 3.932745995 |
| *GTF2B* | 5.49360259 | 3.931507946 |
| *RRM1* | 5.527227513 | 3.927449352 |
| *RER1* | 7.605111304 | 3.925842974 |
| *PLOD1* | 6.875288089 | 3.919188334 |
| *CHAT* | 7.55249332 | 3.917339799 |
| *PHACTR4* | 8.314252347 | 3.917262574 |
| *HRAS* | 2.787255759 | 3.903942401 |
| *ALDH18A1* | 6.417949022 | 3.903879298 |
| *KCNQ1* | 7.224522932 | 3.903714211 |
| *QSER1* | 8.517193191 | 3.90083288 |
| *INADL* | 8.808883285 | 3.900029511 |
| *UBASH3B* | 8.391001166 | 3.898423574 |
| *SORCS1* | 8.658756756 | 3.896957624 |
| *KCNMA1* | 5.966967076 | 3.894719178 |
| *THY1* | 6.545197672 | 3.893786511 |
| *FCGR2B* | 6.472084329 | 3.892733185 |
| *OR9Q1* | 8.282911896 | 3.892241502 |
| *SFN* | 3.730535129 | 3.875022968 |
| *DDOST* | 5.687810705 | 3.87171424 |
| *KCNH1* | 7.093482629 | 3.86663233 |
| *PRCP* | 7.879823026 | 3.86516258 |
| *DISC1* | 7.153527948 | 3.863572999 |
| *SLC1A2* | 6.262748474 | 3.862055964 |
| *ENO1* | 4.006883286 | 3.861739337 |
| *TCP11L1* | 8.517193191 | 3.858224466 |
| *CD2* | 5.830366627 | 3.852662378 |
| *NAV2* | 8.478934479 | 3.848247031 |
| *RPTN* | 6.210616077 | 3.847997422 |
| *PDGFD* | 8.137045891 | 3.844339624 |
| *PHF21A* | 7.367570982 | 3.836343258 |
| *ADIPOR1* | 7.359054923 | 3.832615318 |
| *CTSC* | 6.749897194 | 3.830513263 |
| *FPGT-TNNI3K* | 8.484242091 | 3.829208732 |
| *TRNAU1AP* | 8.314252347 | 3.828494829 |
| *CAMK1D* | 8.185733497 | 3.827780695 |
| *YBX1* | 5.491416797 | 3.824257749 |
| *KCNA4* | 7.631361667 | 3.821495956 |
| *LMO1* | 7.569016271 | 3.810706945 |
| *CDK1* | 2.895525013 | 3.808869826 |
| *CTPS1* | 5.211956201 | 3.808581378 |
| *PDCD4* | 7.006692027 | 3.80847741 |
| *CTRC* | 6.174426309 | 3.807062835 |
| *PLEKHM2* | 7.965033704 | 3.804239328 |
| *KLF6* | 6.528634146 | 3.800029675 |
| *EPRS* | 4.977684194 | 3.794025326 |
| *CEP170* | 5.384747095 | 3.784086262 |
| *GPR37L1* | 7.612570264 | 3.781348277 |
| *CTSS* | 5.596453897 | 3.777983473 |
| *SLC18A3* | 7.55249332 | 3.776160304 |
| *GRM5* | 5.806812375 | 3.775982769 |
| *PAX6* | 5.407685904 | 3.775373154 |
| *TNNI3K* | 8.484242091 | 3.774316869 |
| *TMEM132A* | 7.997506939 | 3.767685809 |
| *SPEN* | 6.450963544 | 3.766555026 |
| *CAT* | 4.853887988 | 3.764952446 |
| *MICALCL* | 8.507242861 | 3.762697898 |
| *LDLRAD3* | 8.527243527 | 3.761503608 |
| *ILK* | 5.000140557 | 3.758947792 |
| *CTNND1* | 5.544217905 | 3.757583487 |
| *FCGR3B* | 6.472084329 | 3.755009473 |
| *ZP1* | 7.997506939 | 3.749460073 |
| *OR51A2* | 7.699397706 | 3.748858359 |
| *ARID5B* | 4.832823893 | 3.747533143 |
| *FASLG* | 5.654135535 | 3.747254559 |
| *ITIH2* | 6.224153511 | 3.746905406 |
| *OR5B17* | 7.156473081 | 3.745655994 |
| *EPHA2* | 5.046936274 | 3.743639577 |
| *TEAD1* | 7.611963407 | 3.737209251 |
| *HNRNPU* | 4.74477714 | 3.737177428 |
| *PIK3CD* | 5.133311532 | 3.733006696 |
| *CLCNKB* | 6.6648091 | 3.729477042 |
| *DHX9* | 4.540319214 | 3.728622066 |
| *CACHD1* | 8.120096333 | 3.723006575 |
| *ARHGEF10L* | 8.184298776 | 3.713658231 |
| *HPS1* | 7.799109526 | 3.708110078 |
| *NOS1AP* | 5.18445311 | 3.706969651 |
| *OR5B3* | 7.156473081 | 3.706227631 |
| *OR1S2* | 6.79531985 | 3.705356628 |
| *PTPRC* | 6.34244147 | 3.70428586 |
| *VPS26A* | 4.96413346 | 3.69968006 |
| *DUSP10* | 7.151101538 | 3.694622232 |
| *TGFBR3* | 6.850430212 | 3.693569904 |
| *MINPP1* | 6.691032295 | 3.692130551 |
| *DRD2* | 4.34743199 | 3.689331208 |
| *RAB30* | 7.879823026 | 3.685398264 |
| *LPGAT1* | 6.735484058 | 3.679095185 |
| *PRMT3* | 6.229721736 | 3.67138592 |
| *APOC3* | 5.866066138 | 3.67069795 |
| *MS4A10* | 7.914791624 | 3.669091322 |
| *SMNDC1* | 5.394608027 | 3.665864917 |
| *KCNQ4* | 7.356075697 | 3.66575576 |
| *SLC25A34* | 7.965033704 | 3.663977136 |
| *EIF3M* | 6.038556155 | 3.662776323 |
| *GLYAT* | 5.770242841 | 3.657708431 |
| *GNG5* | 5.425469152 | 3.654961659 |
| *PAPSS2* | 6.691032295 | 3.653434896 |
| *TCTN3* | 6.417949022 | 3.650907417 |
| *NTM* | 7.644227584 | 3.648941473 |
| *REN* | 6.43962841 | 3.648859932 |
| *FBXO6* | 5.228418239 | 3.648362941 |
| *CKAP5* | 4.957995407 | 3.648138155 |
| *NEBL* | 7.376479727 | 3.647250595 |
| *MEN1* | 6.05760435 | 3.641417072 |
| *FAM129A* | 7.657261193 | 3.635033512 |
| *RHOG* | 4.193723006 | 3.6346167 |
| *PTEN* | 3.758443917 | 3.63454223 |
| *VAMP3* | 5.535813424 | 3.633727043 |
| *WLS* | 7.354667577 | 3.631061133 |
| *RNPEP* | 7.323876967 | 3.6162712 |
| *SH3PXD2A* | 7.399595603 | 3.614652168 |
| *PYROXD2* | 7.799109526 | 3.613199575 |
| *GNL2* | 5.55398411 | 3.613064054 |
| *CD6* | 6.909256405 | 3.611475537 |
| *GMEB1* | 7.980407466 | 3.610767372 |
| *FBLIM1* | 7.965033704 | 3.610477653 |
| *LAMTOR5* | 6.494983596 | 3.608987751 |
| *ZNHIT6* | 7.226034025 | 3.607798094 |
| *ECHS1* | 4.89191936 | 3.599252838 |
| *HPSE2* | 7.799109526 | 3.597901773 |
| *PPIF* | 6.060171745 | 3.596890621 |
| *CD34* | 4.856198941 | 3.590549516 |
| *LRP5* | 6.727101779 | 3.58853587 |
| *SF1* | 6.05760435 | 3.5861608 |
| *TRIM48* | 5.932818352 | 3.585252093 |
| *EIF3I* | 5.294126162 | 3.583284576 |
| *S100A11* | 6.126597221 | 3.582764617 |
| *MACF1* | 5.909331718 | 3.582000033 |
| *GFRA1* | 7.007244177 | 3.581125794 |
| *RNF2* | 3.819899717 | 3.57973361 |
| *OR51L1* | 7.699397706 | 3.572855182 |
| *CALML3* | 5.00356038 | 3.569164959 |
| *CEP350* | 5.836514187 | 3.567367672 |
| *ADSS* | 6.236342591 | 3.566302087 |
| *JAM3* | 7.494023098 | 3.564114732 |
| *CYB5R1* | 7.359054923 | 3.56367899 |
| *EFEMP2* | 3.763603 | 3.563048325 |
| *KRTAP5-9* | 4.170146276 | 3.562739366 |
| *JAK1* | 3.676160682 | 3.562074634 |
| *P2RY6* | 6.88697274 | 3.561565606 |
| *DDX20* | 5.78185213 | 3.556745207 |
| *LDHA* | 4.795362637 | 3.5556125 |
| *ACTA1* | 3.887330393 | 3.550738687 |
| *GLUL* | 6.160119913 | 3.548916191 |
| *RAB42* | 7.980407466 | 3.54549916 |
| *LGR6* | 7.612570264 | 3.545190745 |
| *RNU11* | 7.980407466 | 3.541401691 |
| *EFCAB14* | 7.305847633 | 3.540670924 |
| *NCAM1* | 6.02771368 | 3.5401481 |
| *ARAP1* | 5.8646557 | 3.539959435 |
| *BBOX1* | 6.959996391 | 3.53884361 |
| *HNRNPR* | 4.407960017 | 3.538467838 |
| *BUB3* | 4.603172183 | 3.537578577 |
| *RBM15* | 6.2963606 | 3.533389518 |
| *RRAS2* | 7.01244935 | 3.531560271 |
| *PTPRF* | 5.457313098 | 3.527690774 |
| *ACTN2* | 4.592253961 | 3.52439392 |
| *BAG3* | 3.946096986 | 3.514254423 |
| *NDUFS8* | 5.111505904 | 3.513974419 |
| *KLHL35* | 7.336233161 | 3.513759167 |
| *CNTN2* | 3.437342828 | 3.513698685 |
| *E2F8* | 7.122062982 | 3.512417036 |
| *ARHGAP21* | 6.947952469 | 3.509122119 |
| *RHOC* | 4.374852431 | 3.508368301 |
| *FPGT* | 6.709724428 | 3.506387295 |
| *SRGAP2* | 5.634629616 | 3.506367365 |
| *ZMIZ1* | 7.134530417 | 3.505945194 |
| *FZD4* | 5.492630536 | 3.50281501 |
| *HSPG2* | 5.177693924 | 3.500371744 |
| *OLFML2B* | 6.803395264 | 3.499862999 |
| *ZCCHC24* | 6.060171745 | 3.488119188 |
| *VIM* | 3.403099189 | 3.486731415 |
| *PPP6R3* | 6.727101779 | 3.484982721 |
| *CDC123* | 5.53606707 | 3.484311072 |
| *DESI2* | 6.96517255 | 3.481994776 |
| *TRABD2B* | 7.894736751 | 3.481819038 |
| *P2RY2* | 6.88697274 | 3.477571867 |
| *BIRC2* | 4.431216879 | 3.475007447 |
| *BUD13* | 7.411274328 | 3.47473372 |
| *SHISA4* | 7.881410328 | 3.471198527 |
| *CR2* | 6.804296571 | 3.47029278 |
| *PPFIA4* | 7.359054923 | 3.468291254 |
| *C10orf53* | 7.55249332 | 3.467274892 |
| *TMCO1* | 6.144615727 | 3.465008842 |
| *ZBTB17* | 6.450963544 | 3.464386776 |
| *GHITM* | 4.981027949 | 3.462259551 |
| *PIGK* | 6.530004009 | 3.4621958 |
| *IGF2* | 4.691599914 | 3.458383774 |
| *OR4A15* | 6.221632713 | 3.457028722 |
| *HTR6* | 4.32678116 | 3.456155371 |
| *HTR3B* | 6.648472681 | 3.45590651 |
| *COL11A1* | 6.40516346 | 3.454765112 |
| *ALDH9A1* | 6.144615727 | 3.454555908 |
| *DIP2C* | 6.455406585 | 3.45322273 |
| *FOXJ3* | 6.305080002 | 3.452341403 |
| *ZNF669* | 6.972934005 | 3.451836119 |
| *IVL* | 3.730118202 | 3.451767653 |
| *NBPF10* | 6.983341266 | 3.448251858 |
| *LCOR* | 6.624081228 | 3.446763094 |
| *RPL22* | 6.049517479 | 3.440022948 |
| *ARHGAP22* | 7.484720705 | 3.438037713 |
| *FAM35A* | 7.225346882 | 3.43614647 |
| *ZPR1* | 6.537571985 | 3.433126588 |
| *MFN2* | 6.875288089 | 3.431017707 |
| *SOAT1* | 5.591615178 | 3.426453799 |
| *LMO4* | 6.643853735 | 3.425647823 |
| *PIP4K2A* | 5.563064468 | 3.423145635 |
| *CCND1* | 4.77477297 | 3.421910115 |
| *NPHP4* | 6.294734143 | 3.421854205 |
| *BOLL* | 0 | 3.420207221 |
| *MGEA5* | 4.67559265 | 3.419538611 |
| *CTNNB1* | 0 | 3.418518679 |
| *TRIM66* | 6.54450202 | 3.416621923 |
| *EFHD2* | 6.924901438 | 3.416056399 |
| *USP6NL* | 7.249963937 | 3.414769543 |
| *DNAJB4* | 6.200212218 | 3.414171083 |
| *ARL8A* | 7.612570264 | 3.412900143 |
| *SLC35A3* | 6.252829311 | 3.411666401 |
| *FGFR2* | 3.593569274 | 3.411385997 |
| *RAB18* | 6.262223952 | 3.411304438 |
| *HSD17B12* | 6.340171321 | 3.410392919 |
| *SMC3* | 3.300628327 | 3.406119802 |
| *RAB38* | 7.312420707 | 3.403074091 |
| *ACOT7* | 5.643911078 | 3.398405173 |
| *MAPK8* | 4.722265958 | 3.396266392 |
| *HSP90AA1* | 0 | 3.390032481 |
| *ARHGEF17* | 6.88697274 | 3.387432005 |
| *HBB* | 6.005563471 | 3.387262905 |
| *CD48* | 4.344345568 | 3.385094616 |
| *TCERG1L* | 7.63985121 | 3.382229732 |
| *ZMYND12* | 6.305080002 | 3.3807486 |
| *ATPAF1* | 7.305847633 | 3.377745358 |
| *GAS2* | 7.492586316 | 3.374185889 |
| *FTH1* | 5.044761009 | 3.371823444 |
| *DUSP12* | 6.241979295 | 3.370464249 |
| *TNNI2* | 6.759335274 | 3.36793854 |
| *MGST3* | 6.144615727 | 3.367123822 |
| *SPRR1B* | 4.682591333 | 3.366875868 |
| *S100A10* | 6.126597221 | 3.366504471 |
| *ATP1A1* | 4.104394898 | 3.364173367 |
| *AKT1* | 0 | 3.36293517 |
| *LHX8* | 4.565949473 | 3.360502286 |
| *PPIE* | 4.89686028 | 3.360023801 |
| *CASP4* | 5.390432655 | 3.355455938 |
| *CD247* | 4.901691207 | 3.351596102 |
| *SDF4* | 5.96229417 | 3.350531526 |
| *WDTC1* | 4.601178165 | 3.350142488 |
| *SVIP* | 7.492586316 | 3.348983149 |
| *PSMA1* | 5.253726483 | 3.346234833 |
| *ACRV1* | 3.22590044 | 3.343874202 |
| *MYEOV* | 7.543388553 | 3.343667308 |
| *TCHH* | 6.210616077 | 3.342169491 |
| *PTGS2* | 4.895388806 | 3.340664228 |
| *CCDC179* | 7.492586316 | 3.338735932 |
| *KDM1A* | 2.697407041 | 3.337759447 |
| *ZNF593* | 5.969703055 | 3.336138709 |
| *EP300* | 0 | 3.33187875 |
| *PSMA3* | 0 | 3.329461301 |
| *BTAF1* | 5.090978047 | 3.325391933 |
| *SPRR2G* | 5.621833892 | 3.323620789 |
| *SSX2IP* | 5.54191876 | 3.323257121 |
| *CTR9* | 3.897627125 | 3.321708702 |
| *FAU* | 4.965566331 | 3.320424919 |
| *NOLC1* | 4.928963145 | 3.320353197 |
| *PRDX1* | 3.467978415 | 3.318471598 |
| *ITIH5* | 6.879167822 | 3.318395069 |
| *SERPING1* | 4.376442256 | 3.317875717 |
| *OR10W1* | 7.138049334 | 3.315373054 |
| *ESRRG* | 6.726267403 | 3.313953232 |
| *OBSCN* | 6.387771717 | 3.313778635 |
| *DDAH1* | 7.226034025 | 3.313152206 |
| *ATF6* | 6.803395264 | 3.311350086 |
| *HIPK1* | 5.815161063 | 3.310913121 |
| *PABPC4* | 4.518075479 | 3.309671238 |
| *MKI67* | 5.698794933 | 3.309583526 |
| *SRSF4* | 5.252961665 | 3.309551858 |
| *USP24* | 5.794911408 | 3.306864674 |
| *LSP1* | 6.759335274 | 3.306731625 |
| *AGT* | 4.305806609 | 3.304755967 |
| *KCNA10* | 5.478401428 | 3.303640948 |
| *PUM1* | 4.430376896 | 3.302858733 |
| *TMEM183A* | 7.359054923 | 3.298695776 |
| *TCIRG1* | 5.111505904 | 3.298117206 |
| *CITED4* | 7.356075697 | 3.296754283 |
| *LRRC32* | 5.515727386 | 3.29175444 |
| *ZCCHC11* | 5.566981433 | 3.291187752 |
| *PFKP* | 4.884884089 | 3.289906616 |
| *RPS24* | 5.818520152 | 3.288219296 |
| *CEP164* | 5.667643428 | 3.286800575 |
| *IKBKE* | 5.634629616 | 3.286711816 |
| *USH2A* | 6.940898498 | 3.283004249 |
| *LBX1* | 4.852734512 | 3.282577109 |
| *AKR1C3* | 6.848543419 | 3.279611893 |
| *CTNNA3* | 6.477922814 | 3.279236202 |
| *SPHAR* | 7.364250558 | 3.276081544 |
| *C10orf107* | 7.139435161 | 3.274600629 |
| *RNF186* | 6.783769299 | 3.274000389 |
| *SORBS1* | 6.417949022 | 3.272188104 |
| *ABCC2* | 5.87314832 | 3.266307657 |
| *GDI2* | 4.581453659 | 3.265374774 |
| *SETDB1* | 5.132633451 | 3.261839548 |
| *EPS15* | 3.277034739 | 3.261021775 |
| *LAD1* | 5.650146925 | 3.26077075 |
| *BTRC* | 3.723470899 | 3.260539735 |
| *FCRLA* | 6.472084329 | 3.258999142 |
| *RABGGTB* | 4.014609594 | 3.258102395 |
| *NODAL* | 5.839602198 | 3.257358272 |
| *EIF3F* | 5.739927921 | 3.257142062 |
| *LRRIQ3* | 6.833575881 | 3.252848908 |
| *MIIP* | 6.875288089 | 3.252059496 |
| *PPP2R5B* | 5.72787027 | 3.251592452 |
| *C1orf100* | 6.236342591 | 3.251068559 |
| *SORL1* | 5.441033669 | 3.249860351 |
| *NBPF12* | 6.983341266 | 3.249807452 |
| *ST6GALNAC5* | 6.530004009 | 3.248628247 |
| *ASTN1* | 6.630881405 | 3.24773721 |
| *CCSAP* | 7.364250558 | 3.246248254 |
| *OR10A3* | 6.924291248 | 3.246233218 |
| *NME7* | 4.588313069 | 3.244593343 |
| *CD53* | 5.041506958 | 3.241831291 |
| *CDC20* | 3.839237336 | 3.24029527 |
| *TMEM183B* | 7.359054923 | 3.240096747 |
| *KIRREL3* | 7.165102152 | 3.239354408 |
| *OR10A5* | 6.34414747 | 3.234726023 |
| *FGR* | 3.402798663 | 3.23383851 |
| *KCNAB2* | 6.049517479 | 3.233385177 |
| *SHC1* | 3.268854195 | 3.231286523 |
| *HSPB7* | 6.6648091 | 3.229851192 |
| *PPAP2B* | 7.333780301 | 3.228998827 |
| *RYR2* | 5.695219244 | 3.223802668 |
| *ACTR1A* | 4.26941249 | 3.222401098 |
| *FYN* | 0 | 3.222241359 |
| *PPIH* | 5.491416797 | 3.220573939 |
| *OTUD3* | 6.783769299 | 3.219684433 |
| *AK2* | 4.010738978 | 3.219567688 |
| *WT1* | 5.586799759 | 3.21900392 |
| *ADAM12* | 6.022336211 | 3.218536103 |
| *EHD1* | 6.05760435 | 3.214313577 |
| *PINK1* | 5.464371951 | 3.213385078 |
| *KIN* | 6.224153511 | 3.213092513 |
| *COL24A1* | 6.74563643 | 3.210565005 |
| *SUCO* | 5.654135535 | 3.208831662 |

###### **Table 5. Functional Enrichment of Top 100-400 NAGA Gene Lists in Focal Adhesion, ECM, and PI3K-Akt Pathways**

| Gene List | Pathway | P-value | Number of genes overlapped |
| --- | --- | --- | --- |
| Top 100 | KEGG Focal Adhesion | 9.37e-05* | 8 of 198 |
|  | KEGG ECM-receptor interaction | .0050* | 4 of 88 |
|  | KEGG PI3k-Akt | 1.49e-08* | 15 of 350 |
| Top 200 | KEGG Focal Adhesion | 7.20e-05* | 11 of 198 |
|  | KEGG ECM-receptor interaction | .0342 | 4 of 88 |
|  | KEGG PI3k-Akt | 1.65e-07* | 19 of 350 |
| Top 300 | KEGG Focal Adhesion | 3.85e-05* | 14 of 198 |
|  | KEGG ECM-receptor interaction | .1027 | 4 of 88 |
|  | KEGG PI3k-Akt | 1.60e-08* | 25 of 350 |
| Top 400 | KEGG Focal Adhesion | 1.02e-06* | 19 of 198 |
|  | KEGG ECM-receptor interaction | .0804 | 5 of 88 |
|  | KEGG PI3k-Akt | 5.89e-09* | 30 of 350 |

All p-values corrected for multiple testing using the Benjamini–Hochberg procedure.

* = Significant at *p* <.05
